# Administration of methylene blue in septic shock: pros and cons

**DOI:** 10.1186/s13054-024-04839-w

**Published:** 2024-02-16

**Authors:** Julian Arias-Ortiz, Jean-Louis Vincent

**Affiliations:** 1 Department of Intensive Care, Calderón Guardia Hospital, Universidad de Costa Rica, San José, Costa Rica; 2 Department of Intensive Care, Erasme Hospital, Université Libre de Bruxelles, Route de Lennik 808, Brussels, Belgium

**Keywords:** Methylene blue, Vasopressors, Septic shock, Norepinephrine, Nitric oxide

## Abstract

Septic shock typically requires the administration of vasopressors. Adrenergic agents remain the first choice, namely norepinephrine. However, their use to counteract life-threatening hypotension comes with potential adverse effects, so that non-adrenergic vasopressors may also be considered. The use of agents that act through different mechanisms may also provide an advantage. Nitric oxide (NO) is the main driver of the vasodilation that leads to hypotension in septic shock, so several agents have been tested to counteract its effects. The use of non-selective NO synthase inhibitors has been of questionable benefit. Methylene blue, an inhibitor of soluble guanylate cyclase, an important enzyme involved in the NO signaling pathway in the vascular smooth muscle cell, has also been proposed. However, more than 25 years since the first clinical evaluation of MB administration in septic shock, the safety and benefits of its use are still not fully established, and it should not be used routinely in clinical practice until further evidence of its efficacy is available.

## Background

Sepsis is a common condition that affects millions of people around the world every year [1]. Septic shock, the most severe form of sepsis, is associated with mortality rates of 35 to 50% [2, 3]. Septic shock typically requires the administration of vasopressors, and this treatment should be started without delay to avoid severe hypotension.

In current clinical practice, adrenergic agents are used as the first line vasopressors, acting to rapidly increase vascular tone and blood pressure (through α-1 adrenergic receptors). In addition, they have a short half-life, which facilitates dose titration. However, catecholamines have potential adverse effects. Adrenergic agents can induce myocardial ischemia and decrease regional blood flow; they also have metabolic effects, including causing hyperglycemia and increased cellular metabolism that can participate in the development of hyperlactatemia [4], and can exert immunomodulating effects [5]. Norepinephrine is preferred as the first-choice vasopressor agent, because of its balanced pharmacodynamic profile with potent α-1 adrenergic receptor stimulation and modest β-1 adrenergic effects that can help to maintain cardiac output. Nevertheless, excessive vasoconstriction may increase ventricular afterload thus impairing right and left ventricular function, interfere with regional blood flow distribution, and alter the microcirculation [6].

## Nitric oxide inhibitors

Given these unwanted consequences of adrenergic agent use and the possible advantages of other molecules, the terms decatecholaminization, catecholamine sparing, and multimodal vasopressor strategy have emerged [6–8], which consider the place of non-adrenergic vasopressors in the management of septic shock. Nitric oxide (NO) inhibitors, vasopressin and its analogs, high-dose intravenous hydroxocobalamin, and angiotensin II are among the principal non-adrenergic agents that have been proposed in this context. Because of the complex role of NO in sepsis (see later), administration of NO donors has also been suggested, and shown to improve microcirculatory perfusion, reduce platelet aggregation and microthrombus formation, reduce endothelial permeability and improve tissue perfusion in septic shock [9]. In this review, however, we will focus on the pros and cons of NO inhibitors, particularly methylene blue (MB).

In septic shock, there is significant activation of inducible NO synthase (iNOS), mostly in response to endotoxin and pro-inflammatory cytokines [10]. NO is the main driver of vasodilation leading to hypotension and a decreased response to vasoconstrictors [11, 12]. NO regulates leukocyte activity and is also toxic to most bacteria. iNOS is the major source of NO production in sepsis, mainly in the cytoplasm of cells under pro-inflammatory stress, higher by far than the NO synthesis coming from the constitutive isoforms of the enzyme [13]. At the cellular level, NO also behaves as a free radical, interacts with other free radicals to form secondary metabolites such as peroxynitrite (ONOO −), and via similar reactions it can inhibit lipid oxidation products and act as an antioxidant [14]; this free radical scavenging action reduces tissue injury. At the mitochondrial level, NO takes part in important processes including modulation of the mitochondrial electron transport chain [15].

Several agents have been tested as NOS inhibitors, with the hypothesis that blocking or modulating the excessive iNOS activity would be beneficial in this setting. NOS inhibitors may act to reverse the negative pathophysiological effects of NO overproduction on vascular tone and the hyporeactivity to vasopressor agents during sepsis [16, 17]. In the clinical trial setting, the principal agents that have been used in sepsis for this purpose are non-selective NOS inhibitors, such as methylarginine (L-NMMA) and nitroarginine (L-NNA). These agents have been shown to increase systemic vascular resistance and mean arterial pressure (MAP). It is important to remember however that blocking the NO pathway in septic shock is much more than just the effects on macrovascular hemodynamics, myocardial function, and hyporesponsiveness to adrenergic agents. The role of NO in sepsis is essential for the normal microvascular and immune responses to infection; therefore, global inhibition of NO synthesis in septic shock could be deleterious [18]. Its multiple actions probably explain why the expected improvement in some macrocirculatory variables (for example, increase in MAP, SVR) with non-selective NO inhibitors does not translate directly into further clinical benefits, and may be harmful. Indeed, a multicenter, randomized, placebo-controlled trial by Lopez et al. showed that use of L-NMMA in septic shock patients *increased* 28-day mortality from 49 to 59% [19].

MB has a slightly different profile, as it is an inhibitor of the soluble guanylate cyclase enzyme (sGC), whose activation is important in the NO signaling pathway. sGC catalyzes the production of cGMP in response to NO, and cGMP produces vasorelaxation and inhibits proliferation of vascular smooth cells [20]. Blocking sGC with MB can therefore counteract the hemodynamic effects of NO. Compared with non-selective NOS inhibitors, MB may therefore block some of the deleterious effects of NO, while potentially maintaining its beneficial ones [21]. Use of MB has thus been proposed in septic shock, as in other forms of vasoplegic shock, mainly cardiac surgery, anaphylactic shock, or in reperfusion syndrome after liver transplantation [22].

MB is a non-expensive and widely available molecule, which has been most commonly used to treat severe methemoglobinemia in the context of poisoning. When administered intravenously, MB has an onset of action of 30–60 min with a terminal plasma half-life of 5–6 h [23, 24]. It is metabolized by the liver and excreted primarily by the kidneys, so that patients with dysfunction of these organs have a higher risk of toxicity and drug interactions through cytochrome P450 inhibition [23]. MB is generally well tolerated and toxicity is dose related; blue-green discoloration of urine, skin, and secretions is commonly described, which can interfere with the accuracy of pulse oximeter readings. More severe adverse effects, such as mesenteric vasoconstriction and paradoxical methemoglobinemia, have been reported with higher doses up to 4 mg/kg [24]. Inhibition of monoamine oxidase A by MB can induce a serotonin syndrome. The use of MB is contraindicated in pregnancy and in patients with known glucose-6-phosphate dehydrogenase deficiency.

To date, most studies on the use of MB in septic shock have been observational, with small, highly heterogeneous sample sizes and poor methodologic quality, limiting the conclusions that can be drawn regarding best clinical practice in this context. More than 25 years ago, Preiser et al. reported the hemodynamic effects of MB in 14 patients with septic shock [25]. The authors observed an increase in MAP, associated with an increase in left ventricular stroke work (LVSW) from 42.5 ± 17.9 g/m to 48.9 ± 14.5 g/m, indicating that myocardial function was well preserved. Other investigators reported that MB infusion increased MAP, prevented the decrease in stroke volume and left ventricular stroke work index, and was associated with some reduction in the need for standard vasopressor agents [26, 27]. However, enthusiasm for use of all NO inhibitors in septic shock rapidly decreased after the results of the L-NMMA trial, mentioned earlier, were published in 2004 [19]. Nevertheless, given the issues associated with adrenergic vasopressors discussed earlier, there is renewed interest in the potential role of MB in these patients.

In a recent retrospective cohort study [28], the effects of different dosing strategies of MB (bolus, bolus + infusion, infusion only) were assessed in 209 patients with different types of shock (septic, cardiogenic, vasoplegic) who were receiving norepinephrine at doses > 0.1 mcg/kg/min and had lactate concentrations > 2 mmol/L. Survival was improved in the group receiving a bolus + infusion strategy. In a recent randomized, controlled and double-blinded trial [29], Ibarra-Estrada et al. assessed whether early adjunctive MB administration could reduce the time to vasopressor discontinuation in 92 patients with septic shock who were randomly assigned, within the first 24 h after the start of norepinephrine, to receive an intravenous (IV) infusion of either normal saline or MB once daily for a total of 3 doses. MB-treated patients had a shorter time to vasopressor discontinuation (69 h [IQR 59–83] vs 94 h [IQR 74–141]; *p* < 0.001), more vasopressor-free days at 28-days (*p* = 0.008), a shorter ICU length of stay by 1.5 days (*p* = 0.039), and a shorter hospital length of stay by 2.7 days (*p* = 0.027) than the control group. However, the study had some limitations, including that the associated green discoloration of the urine makes blinding of the study impossible. It would also have been interesting to have more information on cardiac function and on the potential benefit for the patient of reducing the time of exposure to high doses of norepinephrine.

Several systematic reviews or meta-analyses of MB studies in shock have also been published, but all are based on very limited data. In a systematic literature review published in 2010, which included 11 studies (8 observational) in which MB was used for the treatment of septic shock, the authors concluded that MB was associated with short-term improvement in hemodynamic parameters, but little or no benefit on clinical outcomes [30]. They highlighted that in all of the studies MB was administered late in the course of the disease, with important variations in the dose range (1–4 mg/kg) and administration strategy (bolus, infusion, bolus + infusion, for example) across studies. In a meta-analysis of 15 studies in patients with vasodilatory shock, published in 2022, use of MB was associated with a reduction in mortality, but it is important to point out that this study pooled observational and RCT studies, with patients from different etiologies of vasoplegia (surgery, septic shock, etc.) [31]. Two recent meta-analyses included the study by Ibarra-Estrada et al. [24]. In a meta-analysis of 11 randomized controlled trials in perioperative or critically ill patients, Pruna et al. [32] suggested that MB administration may be associated with improved hemodynamics, reduced ICU and hospital lengths of stay, and lower mortality (relative risk 0.60 [95% CI, 0.43–0.84]; *p* = 0.003), while in a meta-analysis of 6 randomized controlled trials assessing MB use in patients with distributive shock, Huang et al. reported reduced duration of mechanical ventilation and hospital length of stay in MB-treated patients, but no effect on mortality [33]. The authors of these meta-analyses highlighted the limitations due to the small population sizes and heterogeneity of the included studies.

## Conclusion

The potential benefits of using agents that act on NO pathways in septic shock is still unclear. Although most evidence comes from small studies and remains largely anecdotal, the use of non-selective NO inhibitors (e.g., L-NMMA and L-NNA) in septic shock has not been shown to provide any substantial benefit in patients with septic shock and use of an iNOS inhibitor increased mortality. MB clearly increases vascular tone and MAP, and seems to maintain cardiac contractility, but without evidence of an increase in cellular oxygen availability. Apart from a consistent reduction in vasopressor requirements with the use of MB, no significant clinically relevant benefit has been demonstrated. The decreased doses of adrenergic agents may be beneficial by reducing the unwanted adverse effects of these agents, but this remains hypothetical as surrogate outcomes, such as time to shock reversal and adverse effect profiles, have not been included in most studies. To elucidate the best timing, dosage, and administration strategy for MB in septic shock patients and finally clarify whether the use of MB really has beneficial or detrimental clinically relevant effects (Table 1), a large multicenter randomized controlled trial should be considered, perhaps using the same protocol as that in the recent study by Ibarra-Estrada et al. [22], in which MB administration appeared safe and effective. Despite some positive findings in recent studies, the significant limitations of the available data, given the small sample sizes and heterogeneity, warrant further assessment of safety and some consistent evidence of advantageous clinical benefit before routine use of MB in septic shock can be considered.

**Table 1:** Use of methylene blue in septic shock: pros and cons

PROS	CONS
Different mechanism of action than adrenergic agents (blocks sGC activity)	Neutral effect or increased mortality in clinical trials of non-selective NO inhibitors in septic shock
Effectively increases vascular tone and arterial pressure	Other agents can also achieve this
Enables other vasopressor (norepinephrine) doses to be reduced	Lack of evidence that this is truly a beneficial effect
Easy application with a continuous infusion	No clear understanding of dosage and timing of administration in relation to norepinephrine administration
Maintains cardiac contractility	Lack of increase in cardiac output and oxygen delivery
Low-cost	Catecholamines are quite cheap too
Widely available	No clear safety profile

sGC: soluble guanylate cyclase

## Data Availability

Not applicable.

## Abbreviations

iNOS: Inducible nitric oxide synthase
MAP: Mean arterial pressure
MB: Methylene blue
NO: Nitric oxide
sGC: Soluble guanylate cyclase enzyme (sGC)
